# Simultaneous Accelerated Corneal Crosslinking and Laser *In situ* Keratomileusis for the Treatment of High Myopia in Asian Eyes

**DOI:** 10.2174/1874364101812010143

**Published:** 2018-07-23

**Authors:** Jin Rong Low, Li Lim, Jane Chwee Wah Koh, Daniel Kai Peng Chua, Mohamad Rosman

**Affiliations:** Singapore National Eye Centre, 11 Third Hospital Avenue, Singapore 168751, Singapore

**Keywords:** Corneal crosslinking, Laser *in situ* keratomileusis, High myopia, Refractive surgery, Keratorefractive surgery, LASIK Xtra

## Abstract

**Background:**

LASIK Xtra is a recently described technique which combines LASIK and accelerated corneal cross-linking(CXL) in the same setting. Its long-term outcome in Asians with high myopia is not well described.

**Objectives:**

To compare the efficacy, predictability and safety of LASIK Xtra with LASIK in patients with high myopia.

**Method:**

This is a retrospective study comparing 50 consecutive eyes undergoing LASIK Xtra for the correction of high myopia and/or myopic astigmatism (-6.63 to -15.50 D manifest spherical equivalent) with a matched control group of 50 eyes undergoing LASIK alone for correction of high myopia (-6.00 to -12.25 D manifest spherical equivalent). Mean follow-up was 5.7 months (range, 1.5-13.3 months) for LASIK Xtra and 3.6 months (range, 1.7-4.2 months) for LASIK only. Outcome measures included Uncorrected Distance Visual Acuity (UDVA), Corrected Distance Visual Acuity (CDVA), refraction and intraoperative and postoperative complications.

**Results:**

At post-operative 3 months, all eyes achieved UDVA of 20/40 or better, and 80.0% of LASIK Xtra eyes achieved UDVA of 20/20 or better, compared to 66.0% of LASIK only eyes (*p* = 0.115). Efficacy indices were 0.99±0.17 for LASIK Xtra and 0.94±0.17 for LASIK only (*p* = 0.164). The proportion of eyes within ±0.50 D of attempted correction was 84% in the LASIK only group and 72% in the LASIK Xtra group at post-operative 3 months (*p* = 0.148). Safety indices were 1.11±0.19 and 1.11±0.18 in the LASIK Xtra and LASIK only groups, respectively (*p* = 0.735).

**Conclusion:**

LASIK Xtra achieved comparable safety, predictability and efficacy as LASIK in patients with high myopia. Good refractive stability was attained at 6-12 months. Further long term studies are required to determine whether simultaneous CXL is able to reduce postoperative LASIK keratectasia in high-risk individuals.

## 1 INTRODUCTION

Laser *in situ* Keratomileusis (LASIK) is the most commonly performed keratorefractive surgery for the correction of ametropia. It results in faster visual recovery and causes less discomfort compared to surface ablation techniques such as photorefractive keratectomy [1].

LASIK keratectasia is a rare but serious complication of the LASIK procedure. Although the mechanisms of postoperative LASIK keratectasia remain unclear, LASIK may reduce corneal biomechanical stability by severing structural lamellae in the anterior cornea during creation of the LASIK flap and removing additional structural lamellae during laser ablation, leading to this complication [2]. Risk factors for postoperative LASIK keratectasia include high myopic corrections, thin corneas and low residual corneal bed thickness [3].

Treatment options for postoperative LASIK keratectasia include rigid gas permeable contact lenses, intrastromal corneal ring segments, and penetrating or lamellar keratoplasty [4]. Corneal collagen cross-linking (CXL), which has been shown to be effective in halting the progression of keratoconus [5], has recently been reported to be successful in slowing or stopping postoperative LASIK keratectasia progression [6-9]. Crosslinking causes photopolymerization of corneal stromal fibers by the combined action of a photosensitizing substance (riboflavin) and Ultraviolet-A (UVA) light. This results in formation of new intrafibrillar and interfibrillar covalent bonds among stromal collagen fibers, which increases the rigidity of the corneal collagen and its resistance to deformation [10]. Accelerated CXL, which utilizes increased irradiation intensity, may be a better alternative to standard CXL for it shortens the procedure time and provides more patient comfort [11].

LASIK Xtra is a recently described technique which combines LASIK and accelerated CXL in the same setting. There are limited studies in the literature which evaluate the outcomes of LASIK Xtra and even fewer that evaluate long-term outcomes in Asians with high myopia. A few studies have reported that LASIK Xtra may confer additional benefits of early refractive and keratometric stability after LASIK, improving the predictability of refractive outcomes in patients with high myopia [12-14]. In this study, we aim to compare the efficacy, predictability and safety of LASIK Xtra with LASIK in Asians with high myopia.

## 2 MATERIAL AND METHODS

In this retrospective study, 50 consecutive eyes of 26 patients undergoing LASIK Xtra for the correction of high myopia and/or myopic astigmatism, defined as myopia greater than -6.00 diopters (D) of spherical equivalent, were recruited at the Laser Vision Centre, Singapore National Eye Centre (SNEC), from April 2013 to December 2015. These were compared with a matched group of 50 eyes of 36 patients which had undergone LASIK alone for the correction of high myopia (greater than -6.00 D) during the same period. Patients returned for 1 day, 1 week, 1 month, and 3 month follow-up after the initial procedure for both the LASIK only and LASIK Xtra groups. A longer follow-up was obtained for the LASIK Xtra group. A comprehensive 100% clinical audit of all LASIK and LASIK Xtra cases in SNEC was performed independently by our Clinical Audit Department. The study was approved by the Sing Health Centralised Institutional Review Board.

Inclusion criteria for surgery were no soft contact lens wear for 1 week before surgery and rigid contact lens wear for 3 weeks prior; stable refractive error for 12 months before surgery; normal peripheral retina or after prophylactic treatment with photocoagulation; no previous ocular surgery, no corneal diseases, no glaucoma; and no history of ocular trauma. Exclusion criteria for surgery were keratoconus or forme fruste keratoconus as evidenced by corneal topography, active ocular or systemic disease likely to affect corneal wound healing, pregnancy, or nursing females. Patients requiring monovision correction were also excluded.

All cases were performed by two surgeons (LL and MR). The cases had a 100-110μm LASIK flap created with the IntraLase IFS femtosecond laser (Abbott Medical Optics, Irvine, CA) and LASIK ablation using either the WaveLight EX500 (Alcon Laboratories, Fort Worth, TX) or WaveLight Allegretto Wave Eye-Q 400Hz (Alcon Laboratories, Fort Worth, TX), wavefront optimised profile. The nomogram supplied by the WaveLight EX500 was used for all cases of LASIK and LASIK Xtra, with a refractive target of plano to +0.5 D for all cases.

Pre-operatively, we evaluated Uncorrected Distance Visual Acuity (UDVA), Corrected Distance Visual Acuity (CDVA), subjective refraction, corneal topography on Orbscan II corneal topography system (Bausch & Lomb Surgical, Orbtek Inc, Salt Lake City, Utah, USA), and Endothelial Cell Counts (ECC) (Konan Medical Corporation, Hyogo, Japan) (only for LASIK Xtra eyes). Analyses were performed for all cases at post-operative 3 months, and for LASIK Xtra cases at post-operative 6 to 12 months.

The efficacy of the surgeries was evaluated by computing the proportion of cases which achieved UDVA of 20/40 or better, and 20/20 or better, and the efficacy index (ratio between postoperative UDVA and preoperative CDVA). The predictability of the surgeries was evaluated by computing the proportion of cases within 1.0 D and within 0.5 D of attempted refractive correction. The safety of the surgeries was evaluated by recording any intra-operative or post-operative complications and by computing the proportion of cases with improved or unchanged post-operative CDVA compared to pre-operative CDVA, and the safety index (ratio between post-operative CDVA and pre-operative CDVA).

### 2.1 Surgical Technique

For LASIK Xtra, after completion of the LASIK excimer ablation procedure, VibeX Xtra (Avedro, Inc., Waltham, MA, USA), consisting of 0.22% saline-diluted riboflavin solution, was placed on the bare stromal bed, and left to soak in for 45 seconds. Special care was taken not to allow the riboflavin solution to come into contact with the LASIK flap. After the 45-second riboflavin soak, the stromal bed was copiously irrigated to remove residual VibeX Xtra, and the LASIK flap was repositioned. Following flap repositioning, UVA fluence of 30 mW/cm^2^ was applied for 46 seconds (total energy 1.4 J/cm^2^), provided by the KXL^®^ CXL system (Avedro, Inc., Waltham, MA, USA). These parameters were recommended by Avedro and have also been used in another study [12].

A bandage contact lens was then placed on the ocular surface and the patient was treated with topical moxifloxacin (Vigamox^®^, Alcon Laboratories, Inc, Fort Worth, TX, USA), dexamethasone phosphate 0.1% (Maxidex^®^, Alcon Laboratories, Inc, Fort Worth, TX, USA), and hypromellose 0.3% and dextran 0.1% (Tears Naturale Free^®^, Alcon Laboratories, Fort Worth, TX, USA). Patients were followed up on the first post-operative day and the contact lens was removed during that visit. Further follow-up examinations were performed at the end of week 1 and months 1 and 3 after surgery for both groups. A longer follow-up of 6-12 months was obtained for the LASIK Xtra group.

### 2.2 Statistical Analysis

Data were analysed using Statistical Package for Social Sciences (SPSS) Version 20.0 (IBM SPSS Statistics for Windows, Armonk, NY: IBM Corp., 2011). Fisher’s Exact Test was used to analyse the efficacy and predictability of the surgeries. Independent Sample T-Test and Non-parametric Mann-Whitney Test were used to analyse the spherical equivalent, efficacy index and safety index.

## 3 RESULTS

### 3.1 Baseline Patient Characteristics

The mean age was 31±7 years (range, 22-46 years) and 31±7 years (range, 21-56 years) for the LASIK Xtra group and LASIK only group, respectively. Fifteen (57.7%) patients in the LASIK Xtra group and 21 (58.3%) patients in the LASIK only group were female. The majority of patients in both groups were Chinese (68% in the LASIK Xtra group and 86% in the LASIK only group) and female (58% in both groups). There was no statistically significant difference between the groups in terms of age (*p*=0.236), ethnicity (*p*=0.274), gender (*p*=0.960) and laterality (*p*=0.688).

Myopia ablation treatment was performed on 50 eyes (100%) and 25 eyes (50%) with the Wavelight EX500 (Alcon Laboratories, Fort Worth, TX) for LASIK Xtra and LASIK only groups, respectively. Twenty-five eyes (50%) in the LASIK only group and none in the LASIK Xtra group were treated with the WaveLight Allegretto Wave Eye-Q 400Hz (Alcon Laboratories, Fort Worth, TX).

The mean follow-up was 5.7 months (range, 1.5-13.3 months) for the LASIK Xtra group and 3.6 months (range, 1.7-4.2 months) for the LASIK only group. All eyes had a mean follow-up of 3 months, while 27 eyes in the LASIK Xtra group had mean follow-up of 6-12 months. Comparison of visual outcome between the two groups was performed at 1 and 3 months’ follow-up.

### 3.2 Refraction Spherical Equivalent

**Fig. (1):**
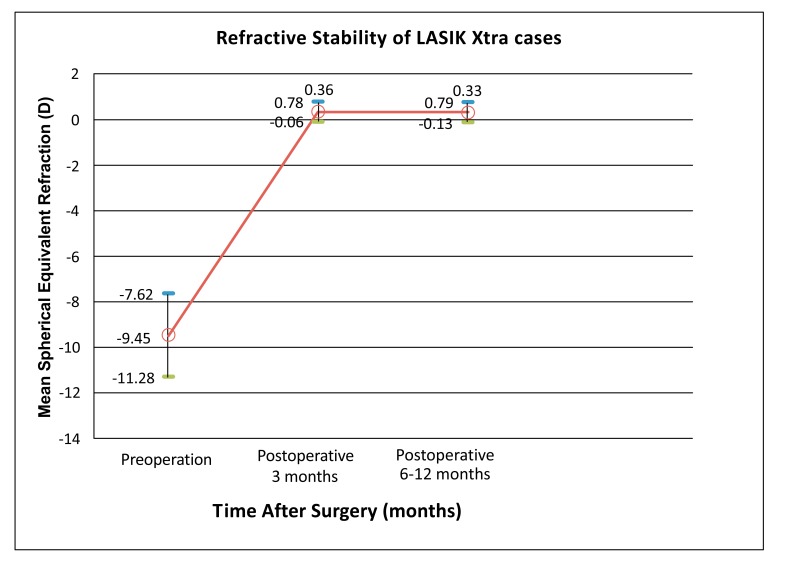
The 6 to12-month stability of spherical equivalent refractive outcome with standard error bars in highly myopic eyes treated with LASIK Xtra.

For the LASIK Xtra group, the pre-operative mean spherical equivalent was -9.45±1.83 D (range, -6.63 to -15.50 D) (Table **1**). The attempted correction mean spherical equivalent was -9.40±1.47 D (range, -6.88 to -13.50 D). The mean spherical equivalent were +0.36±0.42 D (range, -0.50 to +1.25 D) and +0.33±0.46 D (range, -0.38 to +1.25 D) at post-operative 3 months and post-operative 6 to 12 months, respectively. Stability of spherical equivalent refractive outcome at post-operative 6 to 12 months was achieved in the LASIK Xtra group with no regression (*p*=0.243) (Fig. **1**).

**Fig. (2):**
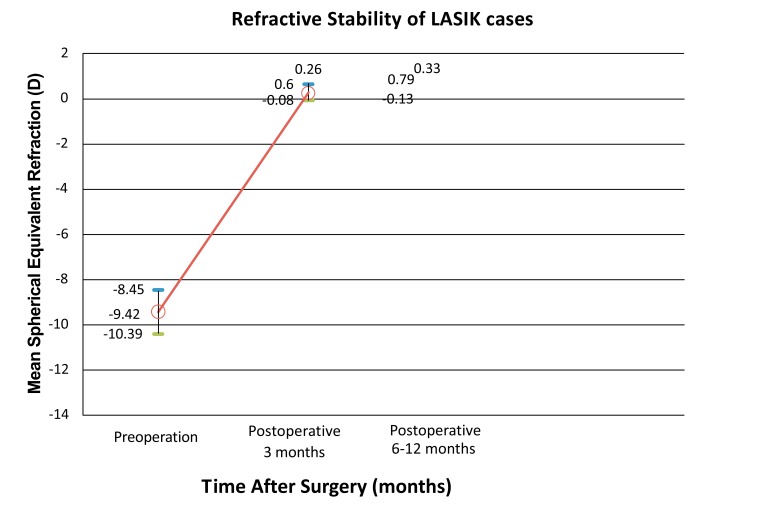
The 3-month stability of spherical equivalent refractive outcome with standard error bars in highly myopic eyes treated with LASIK only.

For the LASIK only group, pre-operative mean spherical equivalent was -9.42±0.97 D (range, -6.00 to -12.25 D). The attempted correction mean spherical equivalent was -9.56±0.86 D (range, -7.00 to -11.63 D). The mean spherical equivalent at post-operative 3 months was +0.26±0.34 D (range, -0.25 to +1.00 D) (Fig. **2**).

There was no statistically significant difference between the LASIK Xtra and LASIK only groups in terms of pre-operative mean spherical equivalent (*p*=0.785) and attempted correction mean spherical equivalent (*p*=0.497). At post-operative 3 months, the mean spherical equivalent were +0.36±0.42 D and +0.26±0.34 D in the LASIK Xtra group and LASIK only group, respectively (*p*=0.243).

### 3.3 Efficacy

**Fig. (3):**
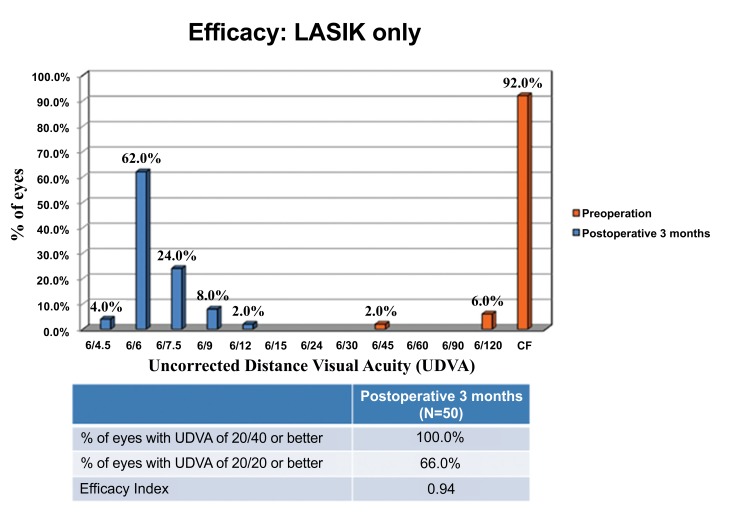
Pre-operative (orange) and 3-month post-operative (light blue) uncorrected distance visual acuity in highly myopic eyes treated with LASIK only.

**Fig. (4):**
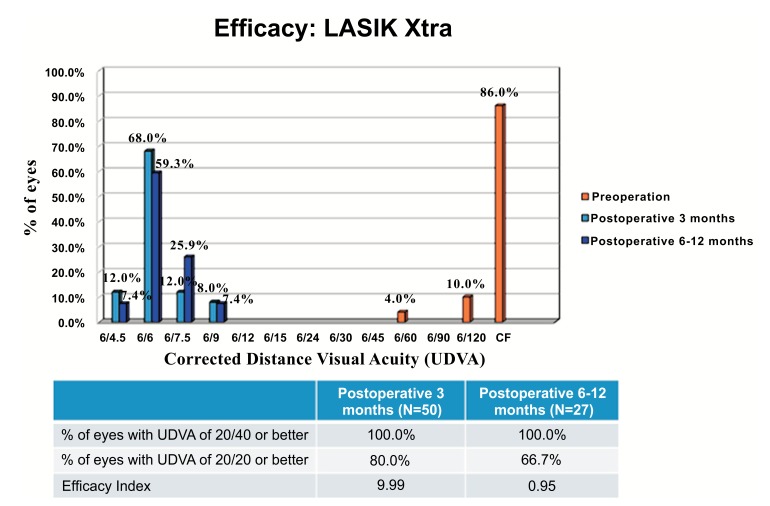
Pre-operative (orange), 3-month post-operative (light blue) and 6 to 12-month post-operative (dark blue) uncorrected distance visual acuity in highly myopic eyes treated with LASIK Xtra.

The proportion of eyes with pre-operative CDVA of 20/20 or better was 98.0% and 96.0% in the LASIK Xtra and LASIK only groups respectively, and this was not statistically significantly different (*p*=1.000). At post-operative 3 months, all eyes achieved UDVA of 20/40 or better, and 80.0% of LASIK Xtra eyes achieved UDVA of 20/20 or better, compared to 66.0% of LASIK only eyes (*p*=0.115) (Figs. **3** and **4**). The efficacy indices were 0.99±0.17 for LASIK Xtra and 0.94±0.17 for LASIK only (*p*=0.164).

At post-operative 6 to 12 months, all LASIK Xtra eyes achieved UDVA of 20/40 or better and 66.7% of them achieved UDVA of 20/20 or better, resulting in an efficacy index of 0.95±0.16.

### 3.4 Predictability

Figs. (**5** and **6**) show the spherical equivalent refractive accuracy at post-operative 3 months for the LASIK only group and at post-operative 3 and 6 to 12 months for the LASIK Xtra group. A greater percentage of eyes were within ±0.50 D of the attempted correction in the LASIK only group (84.0%) than in the LASIK Xtra group (72.0%) at post-operative 3 months (*p*=0.148). Similarly, 100% of eyes in the LASIK only group were within ±1.00 D of the attempted correction, compared to 96.0% of eyes in the LASIK Xtra group (*p*=0.495).

At post-operative 6 to12 months, 95.2% of eyes in the LASIK Xtra group were within ±1.00 D of the attempted correction, and 71.4% of them were within ±0.50 D of the attempted correction.

### 3.5 Safety

**Fig. (7):**
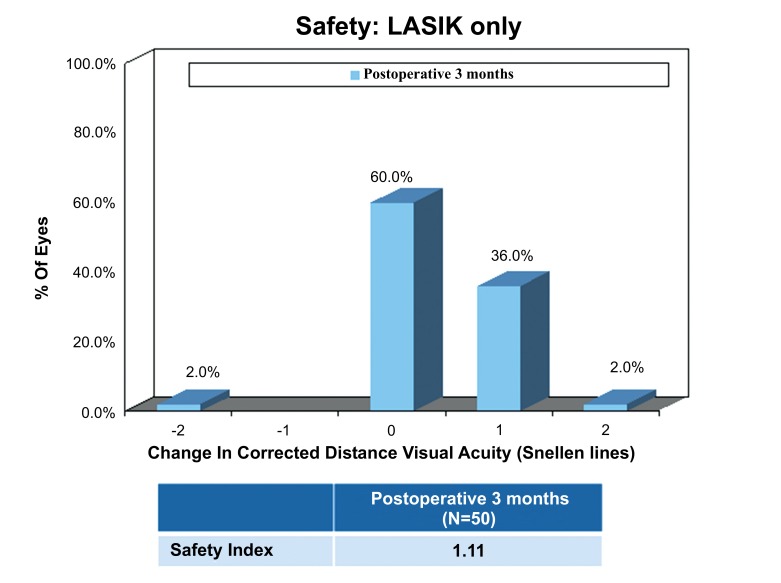
Change in Snellen lines of corrected distance visual acuity in highly myopic eyes treated with LASIK only at post-operative 3 months.

**Fig. (8):**
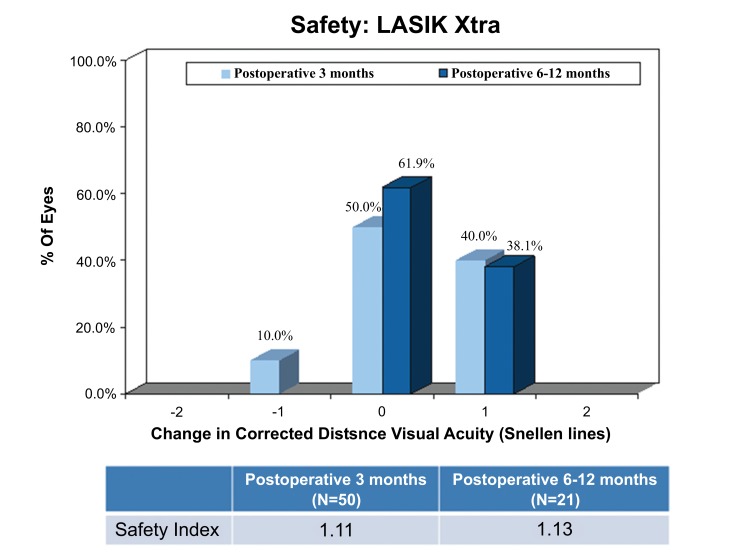
Change in Snellen lines of corrected distance visual acuity in highly myopic eyes treated with LASIK Xtra at post-operative 3 (light blue) and 6-12 months (dark blue).

The safety index was the same in the LASIK Xtra group (1.11±0.19) and the LASIK only group (1.11±0.18) at post-operative 3 months (*p*=0.735). At post-operative 3 months, 20 (40.0%) LASIK Xtra eyes and 18 (36.0%) LASIK only eyes achieved a 1 Snellen line gain in CDVA, while 1 (2.0%) LASIK only eye achieved 2 Snellen lines gain in CDVA. No change in CDVA was found in 25 (50.0%) LASIK Xtra eyes and 30 (60.0%) LASIK only eyes. A decrease in 1 Snellen line of CDVA was found in 5 (10.0%) LASIK Xtra eyes, and a decrease in 2 Snellen lines of CDVA in 1 (2.0%) LASIK only eye (Figs. **7** and **8**).

The safety index was slightly higher at 1.13±0.16 in the LASIK Xtra group at post-operative 6 to 12 months. A gain in 1 Snellen line of CDVA was achieved in 8 (38.1%) LASIK Xtra eyes. There was no Snellen line lost for the LASIK Xtra group at post-operative 6 to 12 months. Post-operative recovery was generally uneventful with no major adverse events observed in either group. Mild post-operative haze was noted in 5 (10.0%) LASIK Xtra eyes, which resolved by the last follow-up visit in 3 of these eyes. 3 of the 5 eyes in the LASIK Xtra group which lost 1 line of CDVA returned for the post-operative 6 months follow-up. Of these, 2 had 1 line of CDVA improvement and 1 remained unchanged compared to the pre-operative CDVA.

Grade 1 diffuse lamellar keratitis was found in 4 (8.0%) LASIK Xtra eyes and all resolved by the second post-operative day. In the LASIK Xtra group, ECC remained stable post-operatively (2893 ± 304), with no statistically significant difference compared to the pre-operative ECC (3010 ± 299) (*p*=0.755) (Table **1**).

## 4 DISCUSSION

The development of progressive postoperative LASIK keratectasia is rare but visually debilitating to the patient. LASIK is challenging in patients with high myopia due to the higher risk of developing post-operative keratectasia and refractive regression. The difficulty of determining who is at marginal risk of postoperative LASIK keratectasia coupled with the regression of LASIK correction over time, particularly in younger patients and those with high myopic corrections, makes it a major challenge. [3, 15, 16] Hence, the use of simultaneous accelerated CXL and LASIK to stabilize the patient’s refraction and cornea immediately after LASIK may be useful, particularly in these high-risk patients.

Long-term studies evaluating the use of CXL to treat keratoconus have shown that it can stabilize keratoconic corneas for more than 3 years. [17, 18] Although we are unaware of any study in literature reporting long term outcomes of LASIK Xtra in Asians with high myopia, one may anticipate that CXL in a non-diseased eye would result in similar stabilization. In a small series of 24 Asian patients with low myopia, Tomita *et al* has reported comparable refractive and keratometric stability in LASIK Xtra and LASIK only at 1 year follow-up [19].

Compared to accelerated CXL for keratoconus which requires a longer riboflavin soak time and utilizes a higher total dose intensity of about 5.4 J/cm^2^ and a longer irradiation time, LASIK Xtra requires a shorter riboflavin soak time of 45 seconds and uses lower parameters with a total dose intensity of 1.4 J/cm^2^ and irradiation time of 46 seconds. The LASIK process provides a natural opportunity for CXL to be performed with minimal interruption to the flow of the procedure. As the LASIK flap is already open, direct application of riboflavin to the corneal stromal bed bypasses the epithelial barrier, thus allowing rapid diffusion of riboflavin into the surrounding stromal tissue. It was found in a study [12] that the addition of CXL to the LASIK procedure did not increase the total procedure time. In our study, the procedure time was increased by 2-3 minutes.

In a prospective study comparing 73 LASIK Xtra eyes and 82 LASIK only eyes, Kanellopoulos *et al* found that 90.4% of LASIK Xtra eyes had UDVA of 20/20 or better as compared to 85.4% of LASIK only eyes at post-operative month 12 (*p*=0.042). [13] Tan **et al**, compared 70 LASIK Xtra eyes and 64 LASIK only eyes in their series of Asian eyes and found that 98% of LASIK Xtra eyes achieved UDVA of 20/25 or better as compared to 61% of LASIK only eyes at post-operative month 3 (*p*<0.001). [12] These findings were similarly shown in a small prospective study comparing LASIK Xtra in one eye and LASIK only in the fellow eye over a 12-month period. [20] In our study, the LASIK Xtra eyes were also found to have superior UDVA at postoperative month 3, and better efficacy index compared to LASIK only eyes, although not statistically significant. While our study groups were matched in terms of baseline patient characteristics, the study by Tan **et al** had a significant difference between the LASIK Xtra and LASIK only groups in terms of age (*p*=0.014). [12] The younger mean age in the LASIK Xtra group (31.29±8.44) compared to that of the LASIK only group (34.56±9) in Tan *et al* ’s study may confound the refractive and keratometric outcomes.

Tan *et al* found that the predictability of the refractive outcomes in the LASIK Xtra group was statistically significantly greater at 3 months, with a greater coefficient of determination and significant reduction in standard deviation of postoperative spherical equivalent in the LASIK Xtra group (*p*=0.005). [12] In our study, the standard deviation of postoperative spherical equivalent was comparable in both groups. Our study found a smaller proportion of LASIK Xtra eyes within ±0.50 D of the attempted correction compared to LASIK only eyes at post-operative month 3, and this decreased slightly at post-operative months 6-12. The lower predictability in the LASIK Xtra group was due to a more hyperopic correction achieved compared to the LASIK only group. This may be due to an additional flattening effect of the cornea from CXL. Although the LASIK Xtra group had a lower predictability, subjects in the group had better visual acuity as compared to those in the LASIK only group due to the hyperopic effect.

Other studies have found that LASIK Xtra may provide the additional advantages of refractive and keratometric stability after LASIK. [12, 13] Kanellopoulos *et al* found a reduced refractive shift in the LASIK Xtra eyes compared to LASIK only eyes, though not statistically significant (*p*=0.063). The mean postoperative Manifest Refraction Spherical Equivalent (MRSE) at postoperative year 1 was -0.19±0.17 D in LASIK Xtra eyes and -0.27±0.23 D in LASIK only eyes. [13] Tan *et al* similarly reported a trend towards reduced refractive drift in LASIK Xtra eyes (-0.04 D) compared to the LASIK only eyes (-0.13 D) (*p*=0.051). [12] Furthermore, Kanellopoulos *et al* reports that corneal keratometry results were stable for LASIK Xtra eyes and slightly regressing in LASIK only eyes (*p*=0.039). [13] Our study revealed a slightly more hyperopic post-operative result in LASIK Xtra eyes (+0.36±0.42 D) compared to LASIK only eyes (+0.15±0.44 D) at post-operative 3 months (Table **1**). However, LASIK Xtra eyes were found to achieve good refractive stability and less regression with spherical equivalent of +0.35±0.45 D at post-operative 6 to 12 months (Fig. **1**). This finding may support the concept postulated by Tan *et al* of a CXL-induced early stabilization of the cornea following corneal wavefront-guided stromal ablation. [12] The lack of longer term follow-up data for the LASIK only group limited comparison between the groups for refractive stability.

The LASIK Xtra group in our study showed a good safety profile. One Snellen line decrease in CDVA was found in 5 (10.0%) LASIK Xtra eyes at post-operative 3 months, which may be due to postoperative corneal haze. The corneal haze in these eyes resolved in 3 out of 5 eyes by post-operative 6 to 12 months (2 eyes lost to follow-up).

Limitations of the study include a relatively high rate of subjects lost to follow-up and short term follow-up period, which is typical of patients undergoing keratorefractive surgery, precluding long-term follow-up. Corneal biomechanical properties were not measured in this study. Corneal hysteresis and corneal resistance factor, measured by the Ocular Response Analyzer (Reichert Ophthalmic Instruments, Buffalo, New York, USA), before and after corneal CXL were found to have no statistically significant difference. [21, 22]

## CONCLUSION

In conclusion, LASIK Xtra achieved comparable visual outcomes compared to LASIK in Asians with high myopia in terms of safety, predictability and efficacy. Good refractive stability was attained at 6-12 months for LASIK Xtra. Further long term studies are required to determine whether simultaneous CXL is able to reduce postoperative LASIK keratectasia in high-risk individuals.

## Figures and Tables

**Fig. (5):**
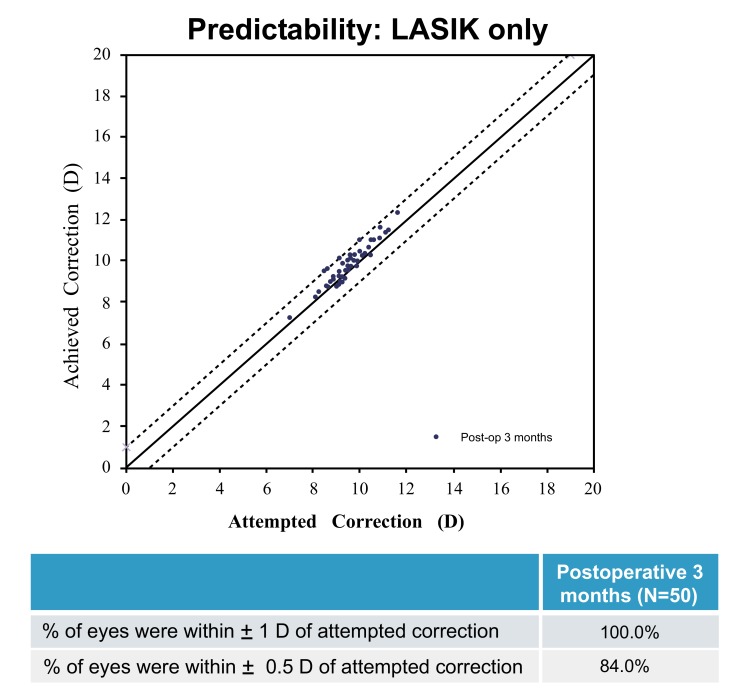
Scatterplot of the spherical equivalent of the attempted correction versus achieved refractive outcome in highly myopic eyes treated with LASIK only at post-operative 3 months.

**Fig. (6):**
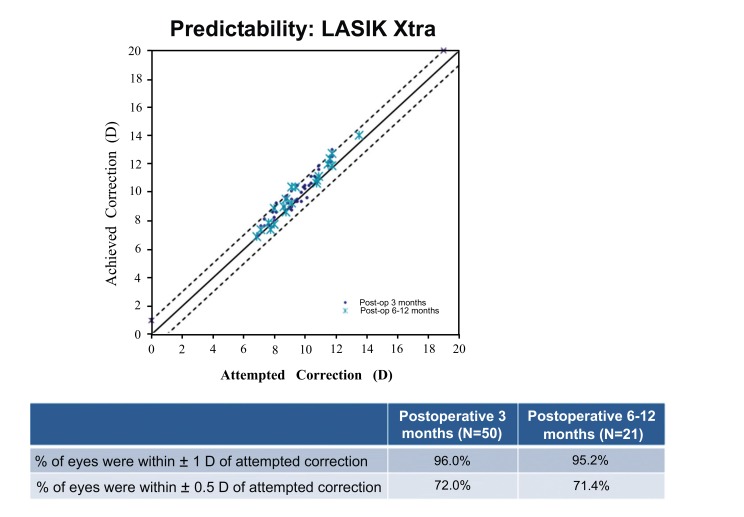
Scatterplot of the spherical equivalent of the attempted correction versus achieved refractive outcome in highly myopic eyes treated with LASIK Xtra at post-operative 3 (dark blue dot) and 6-12 months (light blue cross).

**Table 1:** Pre-operative, attempted and post-operative spherical equivalent refraction for LASIK only and LASIK Xtra groups.

-	LASIK only	LASIK Xtra	*P* value
Pre-operative SE (D)	-9.42 ± 0.97	-9.45 ± 1.83	0.785
Pre-operative range (D)	-6.00 to -12.25	-6.63 to -15.50	
Attempted SE (D)	-9.56 ± 0.86	-9.40 ± 1.47	0.497
Attempted range (D)	-7.00 to -11.63	-6.88 to -13.50	
Post-operative 3 months SE (D)	+0.26 ± 0.34	+0.36 ± 0.42	0.243
Post-operative 3 months range (D)	-0.25 to +1.00	-0.50 to +1.25	
Post-operative 6-12 months SE (D)	-	+0.33 ± 0.46	
Post-operative 6-12month range (D)	-	-0.38 to +1.25	

SE = Spherical equivalent; D = Diopter.

Data are mean ± standard deviation unless otherwise indicated.
